# Percepção de risco e sua influência na vida dos moradores diante da possibilidade de colapso de uma barragem de rejeitos de mineração

**DOI:** 10.1590/0102-311XPT075023

**Published:** 2025-04-28

**Authors:** Hernani Ciro Santana, Renata Bernardes Faria Campos, Michele Corrêa Bertoldi, Julia Silvia Guivant

**Affiliations:** 1 Universidade Vale do Rio Doce, Governador Valadares, Brasil.; 2 Universidade Federal de Juiz de Fora, Juiz de Fora, Brasil.; 3 Universidade Federal de Santa Catarina, Florianópolis, Brasil.

**Keywords:** Efeitos de Desastres na Saúde, Mineração, Colapso Estrutural, Risco, Health Effects of Disasters, Mining, Structure Collapse, Risk, Efectos de Desastres en la Salud, Minería, Colapso de la Estructura, Riesgo

## Abstract

Em 2019, o anúncio da possibilidade de rompimento da barragem Sul Superior, da Mina de Gongo Soco, em Barão de Cocais (Minas Gerais, Brasil), trouxe, e ainda traz, implicações de um colapso real. Este trabalho avaliou a percepção de risco dos moradores diante do risco de ruptura dessa barragem de rejeitos de mineração, além de avaliar a sua influência na vida dos moradores. Entrevistas foram realizadas com moradores do município (n = 62), entre maio e junho de 2021. Dos entrevistados, 38,7% foram removidos de suas residências. Apenas 56,5% ouviram falar sobre o risco do colapso em 2019. As fontes de informações mais usadas para acompanhar a situação de risco foram as redes sociais (45,2%) e a internet (37,1%). Ao receber a notícia, a maioria dos participantes relatou reações de sofrimento e sentimentos negativos, como medo, revolta e desespero; 88,7% relataram o surgimento de problemas econômicos (54,8%), ambientais (41,9%), sociais (32,2%) e de saúde (74,2%), em que ansiedade e depressão foram citadas com maior frequência. Alcoolismo, transtornos psicológicos, úlcera, insônia, arritmia cardíaca e estresse também foram mencionados. A maioria (58,1%) confirmou o agravamento dos problemas de saúde no período da pandemia de COVID-19. Após mais de dois anos do risco anunciado, a crença no rompimento diminuiu entre os entrevistados. Este estudo demonstrou que falhas na comunicação de risco, aliadas à invisibilidade dos efeitos psicológicos e sociais, exacerbaram a vulnerabilidade da população diante dos riscos associados à “lama invisível”. Políticas públicas e ações de saúde voltadas ao cuidado da população são necessárias, principalmente relacionadas ao adoecimento mental.

## Abstract (en)

In 2019, the announcement of the possibility of rupture of the Sul Superior dam of the Gongo Soco Mine in Barão de Cocais (Minas Gerais State, Brazil) brought, and still brings, implications of a real collapse. This study evaluated the risk perception of residents facing the risk of rupture of this mining tailings dam, in addition to evaluating its influence on the lives of residents. Interviews were conducted with residents of the municipality (n = 62) between May and June 2021. Of those interviewed, 38.7% were removed from their homes. Only 56.5% had heard about the risk of collapse in 2019. The most used risk monitoring information sources were social media (45.2%) and the internet (37.1%). Upon receiving the news, most reported reactions of suffering and negative feelings such as fear, revolt and despair; 88.7% reported the emergence of economic (54.8%), environmental (41.9%), social (32.2%) and health (74.2%) problems, of which anxiety and depression were more frequently cited. Alcoholism, psychological disorders, ulcers, insomnia, cardiac arrhythmia and stress were also mentioned. The majority (58.1%) confirmed the worsening of health problems during the COVID-19 pandemic. Over two years after the announcement of the risk, the belief in the rupture decreased among interviewees. This study demonstrated that failures in risk communication, combined with the invisibility of psychological and social effects, exacerbated the vulnerability of the population to the risks associated with the “invisible mud.” Public policies and health actions aimed at caring for the population are necessary, especially those targeting mental illness.

## Resumen

En 2019, el anuncio de la posibilidad del colapso de la presa Sul Superior de la mina Gongo Soco en Barão de Cocais (Minas Gerais, Brasil), ha generado implicaciones de un colapso real. Este estudio evaluó la percepción de riesgo de los residentes ante el riesgo de ruptura de esta presa de residuos mineros y su influencia en la vida de los residentes. Se realizaron entrevistas a los residentes del municipio (n = 62) entre mayo y junio de 2021. De los encuestados, el 38,7% fueron expulsados de sus hogares. Solo el 56,5% de ellos escucharon noticias sobre el riesgo de colapso en 2019. Las fuentes de información más utilizadas para monitorizar la situación de riesgo fueron las redes sociales (45,2%) e internet (37,1%). Al recibir la noticia, la mayoría manifestó reacciones de sufrimiento y sentimientos negativos como miedo, ira y desesperación; el 88,7% tuvieron problemas económicos (54,8%), ambientales (41,9%), sociales (32,2%) y de salud (74,2%), entre los cuales destacan con mayor frecuencia la ansiedad y la depresión. También se mencionaron alcoholismo, trastornos psicológicos, úlcera, insomnio, arritmia cardíaca y estrés. La mayoría (58,1%) confirmó el empeoramiento de los problemas de salud en el período de la pandemia de la COVID-19. Después de más de dos años del anuncio del riesgo, la creencia en el colapso disminuyó entre los encuestados. Este estudio demostró que las fallas en la comunicación de riesgos sumada a la invisibilidad de los efectos psicológicos y sociales intensificaron la vulnerabilidad de la población a los riesgos asociados con el “lodo invisible”. Son necesarias políticas públicas y acciones sanitarias orientadas a la atención de la población, principalmente relacionadas con la enfermedad mental.

## Introdução

Embora essencial para o desenvolvimento econômico, a mineração tem causado mudanças sociais e ambientais em todo o mundo, como doenças, crescimento desordenado, aumento da violência, expropriações e efeitos negativos à biodiversidade ^1^.

Inúmeros problemas com barragens de rejeitos de mineração têm ocorrido em decorrência de manutenção inadequada, falhas mecânicas, deficiências organizacionais, fatores geológicos, fiscalização ineficiente, legislação e responsabilização frágeis, entre outros ^2^ ^,^ ^3^ ^,^ ^4^. Dessa forma, desastres envolvem uma série de atores, que incluem desde as mineradoras até o próprio Estado.

No Brasil, falhas como essas resultaram nos maiores desastres da história da mineração brasileira: os rompimentos das barragens de Fundão (Mariana, Minas Gerais, 2015) e da Mina Córrego do Feijão (Brumadinho, Minas Gerais, 2019). Esses eventos provocaram danos sociais, ambientais, econômicos, culturais, físicos, com violação dos direitos humanos, perdas de vidas, além de danos à saúde dos atingidos bem como de futuras gerações ^3^ ^,^ ^5^ ^,^ ^6^ ^,^ ^7^.

Mesmo tendo se passado vários anos, os efeitos socioeconômicos e ambientais desses desastres ainda persistem e trazem prejuízos à qualidade de vida e à saúde dos atingidos. Esses danos incluem contaminação de recursos hídricos com metais pesados, perda de biodiversidade aquática e poluição, além de doenças (dermatites, diabetes, hipertensão) e efeitos negativos na saúde mental (ansiedade, depressão), instaurando um grave problema de saúde pública no país ^5^ ^,^ ^6^ ^,^ ^7^.

Esse cenário de riscos, doenças e danos configura esses desastres como riscos sistêmicos à sociedade, pois, além de provocarem danos imediatos, estão combinados com outros efeitos negativos de médio e longo prazo, desencadeando reações em cadeia pelo agravamento de doenças pré-existentes e o surgimento de outras novas, com a promoção de riscos de ocorrência complexa e diversificada ^8^. Portanto, do ponto de vista de saúde pública, a compreensão dessas repercussões deve contemplar a identificação e quantificação de novos problemas e necessidades de saúde que surgirão e mobilizarão toda a infraestrutura de saúde pública no sentido de reduzir exposições e riscos, além de cuidar dos danos e das doenças ^9^.

Em 1^o^ de julho de 2024, foram contabilizadas 94 barragens em situação de alerta ou emergência no Brasil, 47 delas em Minas Gerais, sendo três de alto risco (nível de emergência 3): barragem de Rejeitos (Itatiaiuçu), Forquilha III (Ouro Preto) e barragem Sul Superior (Barão de Cocais) ^10^. Estima-se que há em torno de 700 áreas (2.050km^2^) susceptíveis à inundação por rejeitos de barragens de mineração em 178 cidades brasileiras, denominadas manchas de inundação ^11^.

Barão de Cocais encontra-se em uma das situações mais críticas. Das dez barragens de rejeitos de mineração presentes no município, nove delas lançariam rejeitos na área urbana em caso de rompimento ^10^. A barragem Sul Superior, pertencente à Mina de Gongo Soco, teve suas atividades de extração de minério de ferro encerradas pela Companhia Vale em 29 de abril 2016. Após três anos, a situação de alto risco de colapso dessa barragem foi anunciada pela empresa ^12^. A ameaça resultou na evacuação permanente de aproximadamente 500 moradores que residiam na zona de autosalvamento (ZAS), além da remoção temporária de outros 6 mil que estavam localizados na zona secundária de salvamento ^13^. Desde então, somente operações visando à manutenção da estabilidade das estruturas têm sido realizadas no local para minimizar os riscos ^12^.

Barão de Cocais sofre antecipadamente com os transtornos gerados pela possibilidade de rompimento da barragem Sul Superior, situação nomeada pela população como “lama invisível” ^14^. Embora os possíveis danos de um real rompimento sejam preocupantes, a percepção do risco pela população leiga pode não corresponder à realidade desenhada por especialistas, já que ela é construída pela ignorância, por crenças anteriores e experiências subjetivas. Além disso, essas percepções podem ser distorcidas pela maneira como as informações sobre os riscos são expressas e comunicadas ao público. Portanto, é necessário compreender a relação da percepção do risco com a realidade e o contexto político e cultural, para se estabelecer uma comunicação de risco eficaz em situações de desastres ^13^.

Desastre é um processo de perturbação grave do funcionamento de uma comunidade ou de uma sociedade, em qualquer escala, devido a eventos perigosos que interagem com condições de exposição, vulnerabilidade e capacidade, resultando em perdas de vidas e danos materiais, econômicos e/ou ambientais ^15^. Logo, não se restringe às consequências imediatas de um evento ocorrido, pois envolve uma sequência temporal de acontecimentos, associados ao evento em si, com reflexos negativos ao meio ambiente e à sociedade ^8^.

Embora o colapso da barragem Sul Superior ainda não tenha ocorrido, houve mudanças consideráveis na qualidade de vida de muitos moradores. Diante disso, este trabalho objetiva identificar a percepção de risco dos moradores de Barão de Cocais diante da possibilidade de rompimento da barragem Sul Superior; as mudanças na rotina das pessoas atingidas; o nível e a fonte de informações sobre o assunto; e as reações, os sentimentos e os problemas relacionados ao risco anunciado.

Este estudo é oportuno para se discutir a percepção de risco e sua influência na vida de populações de regiões mineradoras em situação de emergência decorrente da possibilidade de colapso de uma barragem de rejeitos de mineração.

## Metodologia

Realizou-se uma pesquisa com 62 moradores de Barão de Cocais, com idade igual ou superior a 18 anos. O cálculo amostral considerou 28.442 habitantes ^16^, nível de 95% de confiança, margem de erro de 12,5% e variabilidade máxima de 50% (p = q = 0,05) ^17^.

As entrevistas foram realizadas entre maio e junho de 2021, mediante aplicação de questionários eletrônicos contendo 33 perguntas (Material Suplementar: https://cadernos.ensp.fiocruz.br/static//arquivo/suppl-e00075023_9454.pdf), com preenchimento pelo aplicativo de gerenciamento de pesquisas Google Forms (https://docs.google.com/forms/u/0/), respeitando o distanciamento social necessário durante a pandemia de COVID-19.

O endereço eletrônico contendo o Termo de Consentimento Livre e Esclarecido (TCLE) e o questionário foi gentilmente enviado aos moradores (e-mail ou WhatsApp) por representantes das associações dos bairros e da Associação dos Moradores Evacuados do Município de Barão de Cocais. No formulário, o respondente somente tinha acesso ao questionário após a leitura do TCLE, de ter declarado ser morador de Barão de Cocais e de ter aceitado participar da pesquisa.

O questionário foi segmentado em três partes: a primeira foi estruturada para coleta de dados socioeconômicos e demográficos; a segunda identificou mudanças na rotina, nível e fonte de informações sobre o assunto, bem como reações, sentimentos e problemas relacionados ao risco de rompimento da barragem; a terceira abordou informações sobre a opinião e as percepções dos moradores a respeito da situação de risco.

Os dados das questões fechadas foram compilados e apresentados de forma descritiva. No caso das questões abertas, foi utilizada a técnica de análise de conteúdo, uma metodologia qualitativa que visa identificar padrões e significados nas narrativas dos participantes. Cada resposta foi lida cuidadosamente, sendo codificada em unidades de significado - trechos ou expressões que captavam a essência das experiências ou dos sentimentos descritos. A partir dessa codificação, foram criadas categorias temáticas, que agruparam os relatos de forma a revelar semelhanças e tendências entre os entrevistados. Entre as categorias emergentes, destacaram-se as percepções de risco, as emoções vividas, as fontes de informação utilizadas e as transformações na vida cotidiana. As respostas das questões abertas mais impactantes foram selecionadas e transcritas.

A pesquisa foi elaborada seguindo as recomendações, normas e diretrizes do Comitê de Ética em Pesquisa (CEP) da Universidade Vale do Rio Doce/Fundação Percival Farquhar (CEP/UNIVALE- 5157) e iniciada após aprovação (20 de abril de 2021, CAAE 45486621.3.0000.5157).

## Resultados

### Perfil sociodemográfico dos entrevistados

**Figura 1:**
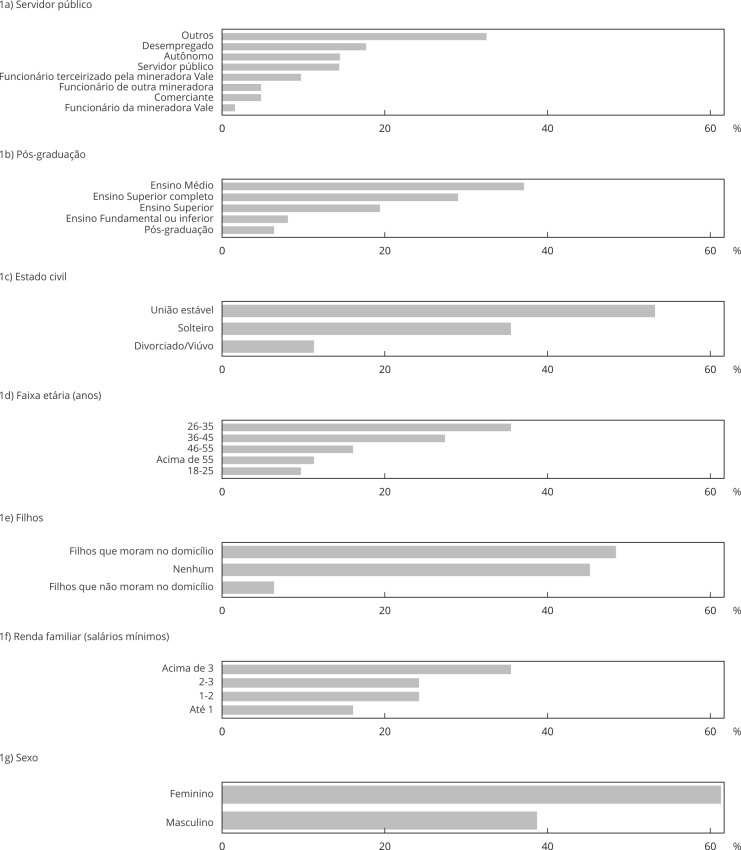
Perfil sociodemográfico dos entrevistados em Barão de Cocais, Minas Gerais, Brasil.

O perfil sociodemográfico dos moradores de Barão de Cocais entrevistados (n = 62) está apresentado na Figura 1. A maior parte era do sexo feminino (61,3%), com idade entre 26 a 45 anos (62,9%), estado civil casado (53,2%), com Ensino Médio completo (56,5%) e renda familiar mensal de um a três salários mínimos (64,5%). Dos entrevistados, apenas 16,1% trabalhavam em atividades relacionadas à mineração, e 17,7% estavam desempregados. Do total, 54,8% tinha filhos; deste grupo, 79,4% tinha um ou dois filhos, e a maioria (88,3% dos entrevistados) residia com os pais, no mesmo domicílio.

A maioria afirmou ser proprietário de imóvel ou lote em Barão de Cocais, enquanto 19,4% alugavam o imóvel em que morava; 37,1% afirmaram que a residência onde moravam se encontrava na mancha de inundação. Do total, 38,7% (n = 24) foram removidos de suas residências pela Vale, devido ao risco de rompimento da barragem Sul Superior. Das famílias evacuadas, 25% tinham residência na mancha de inundação (67% eram proprietários do imóvel) e 62,5% tinham filhos. Mais da metade das famílias evacuadas residia no bairro (comunidade) Socorro (55,2%), enquanto as demais residiam em outros bairros, como Vila do Gongo Soco (17,8%), Tabuleiro (9,3%), Centro (9,4%), Ponte Paixão (4,1%) e Vila Sempre (4,1%). Apenas três respondentes (e suas famílias) foram realocados no mesmo bairro, enquanto os demais foram distribuídos em outros bairros.

### Comunicação de risco

A maioria (79%) dos entrevistados teve conhecimento, pela primeira vez, do tema “rompimento de barragens” em 2015, com o desastre da barragem de Fundão. Outros (6,5%), mais tarde, em 2019, com o desastre em Brumadinho. Poucos (14,5%) tinham conhecimento sobre rompimento de barragens antes de 2015.

Mais da metade (56,5%) ouviu falar, pela primeira vez, sobre a possibilidade de rompimento da barragem Sul Superior, em 2019, enquanto 24,2% tiveram essa informação em 2018; 8,1% souberam desse risco antes de 2018 e 11,3% nunca ouviram falar. As principais fontes utilizadas pelos entrevistados para acessar essa notícia foram a própria mineradora Vale (45,1%), as redes sociais (48,4%) e conhecidos da própria comunidade (25,8%). Fontes relatadas com menor frequência incluíram a televisão (21%), a internet (16,1%), os jornais (16,1%) e o rádio (9,7%).

**Figura 2:**
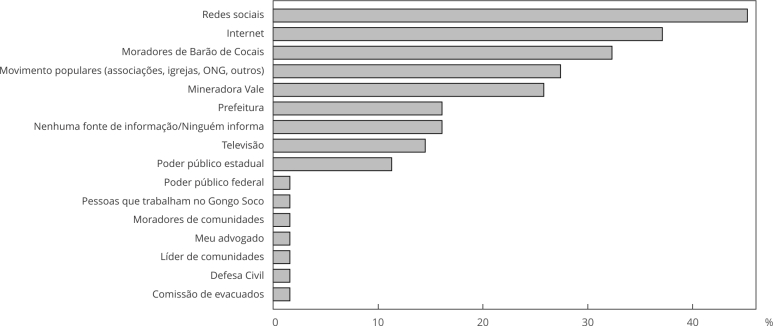
Fontes de orientações e informações sobre a situação da barragem Sul Superior, Barão de Cocais, Minas Gerais, Brasil.

Mais da metade dos entrevistados (62,9%) relatou não receber orientações e informações sobre a situação de risco da barragem Sul Superior, ainda que a maioria (82,3%) tenha buscado essas informações. Apenas 32,3% disseram recebê-las esporadicamente. As fontes de informações mais usadas para esse fim foram as redes sociais (45,2%) e a internet (37,1%). Os moradores do município também fizeram parte da porcentagem de disseminação da notícia (32,3%) (Figura 2).

ONG: organizações não governamentais.

### Percepção de risco

Com a notícia do risco de rompimento da barragem, os sentimentos relatados com maior frequência foram medo (45,2%), revolta (48,4%), desespero (38,7%), raiva (32,5%) e injustiça (38,7%). Preocupação, pânico, angústia, ódio, desconfiança, humilhação, indignação e ansiedade também foram mencionados. As reações imediatas de alguns moradores ao comunicado estão descritas no Quadro 1.

**Quadro 1:** Relatos de alguns entrevistados sobre suas reações imediatas ao receberem, pela primeira vez, a notícia sobre a possibilidade de rompimento da barragem Sul Superior, Barão de Cocais, Minas Gerais, Brasil.

MORADOR	REAÇÃO IMEDIATA DO MORADOR DE BARÃO DE COCAIS DIANTE DA IMINÊNCIA DO DESASTRE ANUNCIADO
A	“*Não acredito em Papai Noel*”
B	“*Fiquei apreensiva. Acabou o meu sossego. E tivemos muitos momentos de pânico e incertezas*”
C	“*Desespero, sensação de que iria morrer atolada na lama, sensação de que iria dormir e não acordar mais*”
D	“*Corri morro acima*”
E	“*Saí de casa correndo, de madrugada, com minhas filhas e a roupa do corpo*”
F	“*Medo de perder o emprego*”
G	“*Fomos retirados de casa com a roupa do corpo, e a minha mãe, com 85 anos de idade, adquiriu, após essa retirada brusca de casa, síndrome do pânico, que a levou a óbito*”
H	“*Triste, mas não acreditei*”
I	“*No terror. Retirar documentos e familiares da casa. E ficar à mercê da sorte*”
J	“*Tentar retirar o máximo de pessoas possível, para tentar salvar a vida dessas pessoas. Por isso, saí chamando todos os vizinhos para ir para um lugar seguro*”
K	“*Preocupei em perder minha terra, sabia que era um golpe. Revolta*”
L	“*Ignorância. Eu acho que isso é apenas um golpe para roubar as terras dos moradores, afinal, fizeram um muro e não serviu para nada*”
M	“*Desespero. Pensando que todos iriam morrer*”
N	“*Extremo desespero. A sensação de medo era avassaladora. Nas primeiras semanas, o sentimento era de total devastação; estávamos todos esperando o pior. Mesmo a minha casa estando fora da mancha, muitos dos meus familiares estavam morando nas áreas de risco*”
O	“*Saí de casa às 3:00 da manhã para aguardar orientações das autoridades competentes*”
P	“*Sair de casa fica no ponto alto*”
Q	“*Medo de não conseguir sair com a minha família a tempo, de não conseguir salvar meus documentos, meus pertences...*”

Além disso, assim que receberam o comunicado, 40,3% acreditaram que o rompimento poderia, de fato, ocorrer. Por outro lado, 45,2% acreditavam que a situação havia sido planejada pela Vale. Passados mais de dois anos, a crença na ruptura da barragem diminuiu; apenas 33,9% a mantiveram.

Metade dos entrevistados não acreditava nas informações e orientações divulgadas pela Vale, enquanto 24,2% acreditavam pouco. Apenas três entrevistados disseram acreditar muito ou totalmente nas informações prestadas pela mineradora.

A Associação de Moradores Atingidos (55%), do Movimento pela Soberania Popular na Mineração (48%) e do Movimento dos Atingidos por Barragens (45%) foram as entidades que mais contribuíram para resolver ou minimizar os problemas decorrentes da situação de emergência da barragem, segundo os entrevistados (Tabela 1). Por outro lado, a maioria dos entrevistados (63% a 84%) declarou receber pouco ou nenhum apoio para garantir a qualidade de vida e segurança no seu bairro de origem, inclusive da própria população (74%) (Tabela 1). A Vale e a prefeitura foram consideradas as que menos contribuíram, em ambos os aspectos (Tabela 1).

**Tabela 1:** Opinião dos entrevistados a respeito do grau de contribuição dos indivíduos ou instituições para resolver ou minimizar os problemas decorrentes da situação da barragem de Gongo Soco, bem como para garantir a qualidade de vida e segurança no seu bairro de origem.

Opinião dos entrevistados a respeito do grau de contribuição dos indivíduos ou instituições para:	Prefeitura (%)	Associação de Moradores Atingidos (%)	População (%)	Igreja (%)	Movimento dos Atingidos por Barragens (%)	Movimento pela Soberania Popular na Mineração (%)	Mineradora Vale (%)
Resolver ou minimizar os problemas decorrentes da situação da barragem de Gongo Soco							
Não contribuem	37	19	21	26	13	18	40
Contribuem pouco	45	26	40	39	42	34	31
Contribuem razoavelmente	11	15	21	21	16	18	13
Contribuem muito	6	23	11	10	16	23	11
Contribuem extremamente	0	18	6	5	13	8	5
Garantir a qualidade de vida e segurança no seu bairro de origem							
Não contribuem	42	29	34	45	37	45	50
Contribuem pouco	37	34	40	26	27	21	34
Contribuem razoavelmente	15	15	15	21	19	18	10
Contribuem muito	6	13	8	8	10	10	0
Contribuem extremamente	0	10	3	0	6	6	6

Os relatos de alguns entrevistados sobre a situação de risco da barragem Sul Superior estão apresentados no Quadro 2.

**Quadro 2:** Comentários de alguns entrevistados sobre a situação de risco da barragem Sul Superior, Barão de Cocais, Minas Gerais, Brasil.

ENTREVISTADO	RELATO DO ENTREVISTADO
A	“*Essa Vale só nos dá nojo. É uma falta de respeito com as comunidades*”
B	“*Gostaria, sinceramente, que algo fosse feito não só por quem foi removido, mas para todos nós que, de alguma forma, tivemos nossas vidas mudadas do avesso de um dia para o outro, somando inúmeros prejuízos, financeiros e emocionais*”
C	“*Minha ação está na justiça e já foi negada. Hoje, eu só quero condições para construir outra casa para minha família em outro bairro, para poder voltar a viver em paz e poder retomar a minha vida normalment*e”
D	“*A Vale sequer indenizou os moradores do meu bairro pelos prejuízos financeiros que acarretou e ainda acarreta, e ela (não os outros atores) deveria ser a principal responsável por isso - e por todas as outras ações*”
E	“*A mineradora mudou a vida de muita gente, sem se importar com sentimentos e sofrimentos, em razão de um lucro que é só dela. Muito triste!*”
F	“*Não vejo responsabilidade de nenhum indivíduo/instituição fora a prefeitura e a população em garantir a segurança do bairro. Acredito que as grandes empresas que atuam na região tenham responsabilidade nisso e não contribuem em nada*”
G	“*Acho uma falta de respeito. Mais de dois anos se passaram e nada se resolveu. Nada se resolveu para os pequenos, porque, para a Vale, o que mudou foi ficar mais rica*”
H	“*Que a mineradora do Gongo Soco deveria pesquisar quantas pessoas ela matou em Barão após o anúncio do rompimento da Sul Superior, em 8 de fevereiro de 2018*”
I	“*Até quando vamos viver nessa situação de terror, medo e angústia? Viver nessa lama invisível é como viver numa guerra fria sem arma. Só Terrorismo. Quero que a população ribeirinha tenha um olhar sem medo, queremos paz e segurança. Não quero viver como uma morta-viva. Pedimos com urgência o descomissionamento da Barragem Sul Superior do Congo Soco*”
J	“*Os poderes públicos não estão cobrando soluções como poderiam cobrar. A Vale está mostrando um interesse muito grande para comprar a comunidade e não cuida da segurança da comunidade. Tivemos coisas roubadas, e nosso maior patrimônio (igreja Nossa Senhora Mae Augusta do Socorro) está sendo destruídos por falta de manutenção. Prefeitura, Vale, MP e Paroquia não fazem nada*”
K	“Sou uma entre as centenas de pessoas, uma vítima da enganação de Vale. Tive meu projeto * impedido e não fui indenizado por isso. A Vale nos enganou e com o apoio da Defesa Civil, que nos fez acreditar na farsa montada para atender seus interesses econômicos”
L	“*Hoje, não consigo plantar minhas verduras, o que me garantia uma renda extra. Vivo dentro de um apartamento. Eu tinha uma empresa de produtos orgânicos, trabalhava dentro das comunidades; hoje, não tenho mais nada. Estou na cidade obrigado. Sou visto como aproveitador de uma situação. Aproveitar o quê?*”
M	“*Barão veio de Socorro, tudo começou aqui. Mas, quem gosta só de dinheiro, não gosta de história, né?!*”
N	“*Ainda tenho fé em Deus de que iremos ter nossa festa da Nossa Mãe Augusta, ela sempre nos uniu e amparou. Isso não pode acabar assim*”
O	“*Eu sei das rotas de fugas, participei dos treinamentos e digo de passagem, esses treinamento deu gente no início, pois todo mundo estava com medo. À medida que passava o tempo, ‘menas’ gente ia neles, “Ocê” viu o pessoal de Brumadinho, que só está vivo porque seguiu o caminho que conhecia? Se fosse no caminho marcado, eles ‘tava’ tudo morto agora*”
P	“*O duro é pensar que quem está com a mão no botão da sirene para salvar a gente é a Vale. E o que a gente vale para a Vale?*”
Q	“*É muito óbvio o interesse da Vale naquela região. Na minha ‘opinião’ essa é a nova estratégia da empresa, que enfrenta várias dificuldades no licenciamento ambiental de projetos na região para conseguir seus objetivos*”

* Descrição removida para inviabilizar a identificação do entrevistado.

### Repercussões na qualidade de vida

A maioria (88,7%) indicou que surgiu pelo menos algum problema após o recebimento da notícia sobre a possibilidade de rompimento da barragem, incluindo problemas de saúde (74,2%), econômicos (54,8%), ambientais (41,9%) e sociais (32,2%).

Quando questionados se algum membro da família, incluindo o próprio respondente, havia apresentado algum problema de saúde no período entre a notícia do risco de rompimento da barragem e a pandemia (março de 2020), 62,9% dos entrevistados afirmaram que sim. Ansiedade e depressão foram os problemas de saúde relatados com maior frequência. Alcoolismo, mudança de humor, bruxismo, déficit de atenção, síndrome do pânico, transtornos psicológicos, úlcera gástrica, insônia, arritmia cardíaca, obesidade, acidente vascular cerebral, hipertensão, estresse, síndrome do coração partido e agravamento de doenças preexistentes também foram mencionados. Além disso, 58,1% dos entrevistados relataram que os problemas de saúde foram agravados durante a pandemia.

Mais da metade dos moradores (56,5%) afirmou que existiam problemas socioambientais associados à mineração no seu bairro de origem (bairro onde residiam antes da evacuação). Uma parte (27,4%), entretanto, teve opinião contrária, negando a existência de tais problemas, enquanto 16,1% não souberam responder. O aumento da insegurança do local, depredação, roubos, destruição das residências, poluição, mato, ruídos e abandono do local foram mudanças no bairro de origem mencionadas pelos entrevistados. Consequências como diminuição do convívio social e com a natureza, mudança de atitudes de moradores, especulação imobiliária, desemprego, escassez de moradias, expansão da mineração no município, inclusive em área urbana, fechamento de comércios e deslocamento do centro comercial também foram relatadas.

## Discussão

### Perfil sociodemográfico dos entrevistados

Segundo dados do último censo demográfico ^18^, Barão de Cocais apresentou população com 51,1% de mulheres e 36,3% com rendimento mensal *per capita* de até meio salário mínimo; apenas 25,9% estavam ocupados, predominantemente na faixa etária entre 30 a 44 anos ^18^. Os dados sociodemográficos apresentados tanto pelo censo quanto pelo presente estudo (Figura 1) refletem uma população economicamente vulnerável, agravada pela instabilidade trazida pelos riscos ambientais e pela insegurança trabalhista, tornando-a mais susceptível aos riscos modernos ^19^.

Além disso, o perfil dos entrevistados evidenciou a vulnerabilidade em relação aos bens materiais, considerando que muitos proprietários de imóveis foram removidos de suas residências pela Vale e, ao mesmo tempo, a fragilidade decorrente da mudança do contexto socioeconômico (afastamento de amigos e familiares, mudanças na rotina, interrupção das fontes de renda como plantações e criação de animais) e ambiental, contextos também relatados por Laurino ^20^ e Lima ^21^.

### Comunicação e percepção de risco

No momento da notícia sobre a possibilidade de rompimento da barragem, sobressaíram sensações negativas, tanto associadas à crença na possível ruptura (medo, pânico, angústia, preocupação e ansiedade) quanto à sua descrença (ódio, desconfiança, humilhação, indignação), confirmadas por relatos de vários moradores (Quadro 1).

Segundo Beck ^19^, indivíduos expostos a riscos constantes tendem a negar a existência ou gravidade deles por meio de um estado de dessensibilização ou habituação, como um mecanismo de defesa psicológica e cultural para evitar ansiedade e estresse contínuo, que influencia a percepção do risco ^19^. Tal percepção também é moldada por fatores sociais e políticos, e varia para diferentes grupos sociais ^19^, o que exige a inclusão de múltiplas partes interessadas para uma gestão eficaz ^14^.

Uma vez que a percepção de risco se trata de um processo tanto cognitivo quanto emocional, a comunicação eficaz e transparente torna-se uma ferramenta essencial para reduzir a incerteza e o medo. A falta de informação clara pode aumentar a ansiedade e, por isso, estratégias eficazes de comunicação, adaptadas às necessidades e contextos culturais das comunidades afetadas, contribuiriam significativamente para minimizar os efeitos negativos na saúde mental da população ^22^.

A confiança na fonte de informação é fundamental para que as mensagens de risco sejam recebidas adequadamente, e, nesse sentido, a confiança na Vale e nas instituições públicas envolvidas era crucial, mas foi enfraquecida por sucessivos desastres e uma comunicação inconsistente. Essa falta de confiança na Vale é retratada neste estudo, uma vez que a maioria dos entrevistados não acreditou no risco de rompimento da barragem, mesmo após dois anos do anúncio. Além disso, relatos de vários moradores (Quadro 2) evidenciaram a insatisfação e a desconfiança em relação às ações da mineradora e do poder público; enquanto 45,2% acreditavam que essa situação havia sido planejada pela mineradora, 50% não acreditavam nas informações e orientações divulgadas pela Vale e a maioria não adotou nem a Vale (74,2%) nem a prefeitura (83,9%) como fonte de informações (Figura 2). Sentimentos similares foram identificados na comunidade de Antônio Pereira, Ouro Preto (Minas Gerais), que passou a sofrer a insegurança do risco de rompimento da barragem Doutor, em 2020, o que resultou na remoção de mais de 500 residentes da ZAS pela Vale; diante da insegurança vivida, várias manifestações públicas foram realizadas para reivindicar à Vale melhorias na comunicação e acesso aos processos de reparação no local ^23^.

Cabe ressaltar que, conforme relatado por Lima ^21^, o risco percebido pela população de Barão de Cocais não se restringe apenas à possibilidade de rompimento da barragem, mas se estende a quaisquer ameaças associadas ao contexto da lama invisível, como os riscos de arrombamentos e furtos que passaram a ocorrer nos territórios, os riscos associados ao novo estilo de vida imposto pela evacuação, que privou a população do convívio anterior, os perigos do cotidiano urbano, o risco de não recuperarem os bens materiais ou de não receberem indenizações, entre outros. Neste estudo, ficou evidente a percepção de abandono e descaso, pois a maioria dos entrevistados afirmou que a mineradora não contribui ou contribui pouco para resolver ou minimizar os problemas decorrentes da situação da barragem de Gongo Soco (71%) e para garantir a qualidade de vida e segurança no seu bairro de origem (84%) (Tabela 1).

A percepção individual do risco associada aos danos de desastres pode ser influenciada também pela falta de credibilidade em informações divulgadas pela mídia, uma vez que podem estar inseridas em discursos contraditórios e norteadas por interesses específicos ^24^ ^,^ ^25^. A cobertura midiática de desastres como o de Mariana frequentemente enfatiza o drama, mas falha em educar o público sobre os processos subjacentes ao risco e as medidas de prevenção ^24^ ^,^ ^25^. Ao analisarem os tensionamentos entre as narrativas da Vale e as das comunidades de Barão de Cocais em coberturas jornalísticas sobre o risco de rompimento da barragem, Carvalho et al. ^26^ consideraram que a cobertura privilegiou as informações da mineradora, configurando, assim, sua hegemonia discursiva e indicando uma invisibilização daqueles mais propensos ou condenados à catástrofe. Portanto, uma comunicação ineficaz pode prejudicar ações populares individuais e coletivas que contribuam para a segurança e o bem-estar social.

De acordo com Beck ^19^, quando as instituições que deveriam proteger a sociedade falham, o risco se torna ainda mais perigoso, porque a sociedade perde confiança em sua capacidade de resposta e proteção. Se as instituições são vistas como confiáveis, as pessoas tendem a aceitar as informações e as medidas de mitigação de risco fornecidas. No entanto, se são percebidas como incompetentes ou corruptas, as pessoas estariam mais propensas a negar os riscos ou adotar medidas próprias de mitigação, pois não confiam nas autoridades para protegê-las ou fornecer informações precisas ^19^.

No dia do simulado de evacuação realizado pela Vale, em Barão de Cocais, a prefeitura decretou feriado municipal para que o comércio não funcionasse, permitindo a participação do maior número de moradores possível. Estava prevista a participação de seis mil moradores ^27^, mas menos de 30% compareceram ^28^ ^,^ ^29^. Esse baixo índice de participação reflete a desconfiança generalizada nas autoridades e na mineradora, conforme mostrado pelo relato do entrevistado O (Quadro 2), indicando que os simulados de emergência, embora necessários, não têm credibilidade o suficiente para engajar a população de forma efetiva. Numa situação real, atitudes como essas teriam resultado em vários óbitos. O baixo índice de participação no simulado de evacuação demonstra como a comunicação falha e a falta de credibilidade da fonte de informação podem aumentar o risco, em vez de mitigá-lo.

Portanto, a comunicação ineficaz e a desconfiança generalizada nas instituições responsáveis pela segurança da população são evidentes em Barão de Cocais, e esse déficit comunicacional colabora para o agravamento da crise de confiança nas autoridades, conforme demonstrado por Laurino ^20^. Segundo Beck ^19^, a falta de confiança nas instituições em tempos de risco gera um ciclo vicioso em que as medidas de prevenção e comunicação são desconsideradas pelas populações afetadas, que, por sua vez, se sentem desamparadas e negligenciadas.

### Repercussões na qualidade de vida

Desde o anúncio do risco de rompimento da barragem, o surgimento de diversos problemas foi relatado pelos entrevistados, incluindo problemas de saúde, econômicos, ambientais, sociais, além de outros relacionados à cultura, ao lazer e à religiosidade. Esses resultados demonstraram claramente que os prejuízos diante do risco são de difícil mensuração em Barão de Cocais, conforme previamente discutido por Gonçalves & Santos ^30^ e Laurino ^20^. Essa “espera constante pelo desastre” aprofunda o trauma da população, gerando um estado de ansiedade crônica e medo.

Muitos dos problemas relatados pelos moradores são similares àqueles ocorridos em regiões nas quais rompimentos de barragens ocorreram, evidenciando que, embora o colapso não tenha acontecido, danos similares emergiram ^7^ ^,^ ^28^. Conforme observado por Laurino ^20^, a situação de risco vivenciada em Barão de Cocais tem causado efeitos negativos similares aos de um desastre, como traumas psicológicos e mudanças de comportamento que se assemelham aos sofridos por vítimas diretas.

De maneira geral, sintomas psiquiátricos e problemas neurológicos (depressão, amnésia, culpa, ansiedade, descontrole emocional, isolamento, medo, memórias intrusivas, solidão, tristeza) são os prejuízos mais frequentes em populações atingidas por desastres com barragens de rejeitos de mineração no Brasil, principalmente na fase de reconstrução ^7^. A saúde mental, nesse contexto, se torna o campo mais vulnerável, já que os traumas se prolongam no tempo, afetando as comunidades muito além dos momentos críticos de crise física. Esses sintomas normalmente vêm acompanhados de outros problemas de saúde (dor de cabeça, náuseas, queda de cabelo, problemas respiratórios, osteoarticulares, gástricos e cardiovasculares, disfunção sexual, diarreia, febre, problemas de pele) que, muitas vezes, estão associados ao adoecimento mental ^7^ ^,^ ^31^.

Uma revisão sistemática realizada com 19 de 157 publicações (PubMed, Scopus e Web of Science) relacionadas aos danos físicos e psicológicos decorrentes de colapsos de barragens de rejeitos de mineração mostrou maior prevalência de efeitos negativos na saúde mental dos atingidos, como ansiedade, depressão, insônia, instabilidade emocional, irritabilidade e transtorno de estresse pós-traumático ^32^. As consequências psicossociais para a população atingida direta e indiretamente pelos desastres em Brumadinho e Mariana também foram compiladas em uma revisão de literatura integrativa realizada com 14 dos 41 artigos encontrados em três bases de dados, entre 2015 e 2020 ^33^. Sintomas depressivos (29,3%) foram a condição mais prevalente entre a população adulta de Brumadinho (2.740 atingidos), seguidos por sintomas de estresse pós-traumático (22,9%) e ansiedade (18,9%), acometendo, principalmente, mulheres e moradores de regiões atingidas pela lama ^34^. A consistência desses dados reforça o caráter sistêmico do sofrimento social e psicológico associado aos desastres de mineração no Brasil, indicando que as intervenções precisam ser contínuas e não apenas emergenciais. Ao mesmo tempo, reforçam a necessidade de uma intervenção integrada à saúde mental e física, especialmente em comunidades que vivem sob constante ameaça de desastres.

Em geral, rompimentos de barragens também têm alterado significativamente a rotina das comunidades afetadas. No Espírito Santo, em cidades como Regência, Povoação e Linhares, a contaminação da foz com rejeitos da barragem de Fundão causou repercussões sociais (conflitos, falta de entretenimento) e prejuízos econômicos (interdição da pesca, redução de atividades comerciais e agrícolas) ^33^. Em Colatina (Espírito Santo), Mariana e Barra Longa (Minas Gerais), danos psicológicos e emocionais surgiram diante das alterações na rotina, além de problemas de saúde ^35^ ^,^ ^36^. Em Governador Valadares (Minas Gerais), foram observadas mudanças nos hábitos alimentares e de consumo de água decorrentes da percepção de risco de contaminação da água e de alimentos com a lama ^37^. Repercussões negativas na identidade cultural foram observadas em Bento Rodrigues (Mariana, Minas Gerais), completamente destruída, com perdas de vidas e da herança cultural e histórica da comunidade. Além do sentimento de desapropriação do próprio futuro decorrente da desterritorialização, o processo de reassentamento dos atingidos em novo local provocou uma ruptura de identidade causada pela percepção de falta de senso de pertencimento ao lugar, além de conflitos sociais ^38^. Prejuízos econômicos desses desastres incluíram a perda da propriedade ou posse do imóvel, da capacidade produtiva da terra, das áreas para fins de exercício físico e recreação e de meios de sustento ^31^.

Esse panorama não se limita apenas ao contexto de desastres com barragens de mineração, mas também à influência da atividade mineradora na saúde das populações que vivem em regiões minerárias, principalmente daquelas que vivem a insegurança de um possível colapso de barragem. Problemas similares ocorreram em Barão de Cocais, relacionados a efeitos psicológicos e socioeconômicos decorrentes da evacuação dos moradores de seus locais de origem, que perderam a posse da terra ou do imóvel, seus meios de geração de renda e sustento, além de sua identidade (Quadro 2). Além disso, perdas imateriais imensuráveis que comprometem a identidade cultural e religiosidade da população foram observadas no município. Nesse contexto, ressalta-se a importância da Igreja Nossa Senhora Mãe Augusta do Socorro (construída em 1737) e da tradicional festa da padroeira Nossa Senhora Mãe Augusta na vida das comunidades, evidenciada pelos relatos dos moradores J e N, respectivamente (Quadro 2). Em Antônio Pereira (Ouro Preto, Minas Gerais), o risco de rompimento da barragem do Doutor também trouxe medo e desconfiança, além de problemas de saúde, ambientais, sociais e culturais decorrentes das obras de descomissionamento da barragem e da remoção de várias famílias da ZAS ^23^. Situação similar foi observada em São Sebastião das Águas Claras (Macacos, Minas Gerais), diante da possibilidade de rompimento da barragem de minério B3/B4, em 2019, em que foram identificados danos ao meio ambiente, bem como violações de direitos, particularmente no que tange aos direitos das crianças (educação, lazer e saúde), com danos a curto e longo prazo ^39^ ^,^ ^40^.

Em um estudo cooperativo entre a Fundação Oswaldo Cruz (FIOCRUZ) e o Movimento dos Atingidos por Barragens (MAB) ^7^, que realizou um levantamento de estudos publicados entre 1940 e 2022 (dez bases de dados científicos) sobre a influência das barragens na saúde e no ambiente dos territórios brasileiros, dos 2.157 trabalhos selecionados pelo estudo, apenas 434 evidenciaram os efeitos negativos à saúde, enquanto a maioria discutiu os danos ambientais e as perdas na biodiversidade. Além de mostrar evidências de que a avaliação da influência da mineração na saúde das pessoas não é priorizada, o estudo concluiu que há uma invisibilização dos processos de vulnerabilidade dos diferentes grupos sociais decorrentes da construção e funcionamento de barragens.

Portanto, a carência de evidências científicas substanciais sobre a influência da mineração na vida das comunidades, principalmente em situações de risco de rompimentos de barragens, dificulta o direcionamento de estratégias de intervenção e ações de saúde voltadas à melhoria da qualidade de vida e saúde da população. Estudos que identifiquem as lacunas no conhecimento dentro da abordagem da saúde pública são de extrema relevância para orientar políticas públicas na área de saúde e soluções viáveis a serem implementadas dentro do Sistema Único de Saúde (SUS) voltadas a reduzir exposições e riscos, além de cuidar dos danos e doenças.

Este estudo demonstra que as falhas na comunicação de risco, aliadas à invisibilidade dos efeitos psicológicos e sociais, exacerbaram a vulnerabilidade da população de Barão de Cocais diante dos riscos associados à barragem Sul Superior.

A aplicação de questionários eletrônicos revelou limitações metodológicas, como a baixa possibilidade de verificação da sinceridade do entrevistado, a dependência do nível educacional para compreender as perguntas, a necessidade de acesso à internet para responder ao questionário e o baixo controle amostral, que podem resultar em vieses na pesquisa. Há limitação na aquisição de informações pelo roteiro de perguntas (quantidade e detalhamento), dificultando a compreensão de alguns aspectos do estudo ^41^. Além disso, não foi possível garantir a isonomia e aleatoriedade na participação dos entrevistados.

Entretanto, o estudo forneceu dados valiosos sobre a percepção de risco e os impactos sociais e econômicos sofridos. Para superar as limitações, estudos futuros devem incluir amostras mais amplas e representativas, além de adotar abordagens qualitativas que permitam compreender as relações complexas entre a percepção de risco e o contexto social. Além disso, estudos qualitativos com moradores expostos a diferentes níveis de risco (residências fora e dentro da Mancha de Inundação) ou grupos específicos (renda, escolaridade, faixa etária) são necessários para melhor compreender a relação entre a percepção de risco e o contexto real, visando a melhoria na comunicação de risco ^14^ ^,^ ^19^ ^,^ ^22^.

Em suma, para que haja uma mitigação eficaz dos efeitos psicológicos e sociais dos riscos de barragens, é necessário que as intervenções sejam contínuas, inclusivas e participativas, garantindo que as populações afetadas sejam não apenas destinatárias, mas também protagonistas das políticas de gestão de riscos.

## Conclusão

Não obstante, felizmente, o colapso da barragem de Gongo Soco não ocorreu até o momento, embora o risco persista e siga impactando negativamente a população de Barão de Cocais. Este trabalho aponta mudanças significativas na qualidade de vida de moradores de Barão de Cocais, além dos prejuízos econômicos, sociais e à saúde da comunidade resultantes do risco de rompimento da barragem Sul Superior.

Os resultados desta pesquisa evidenciam a necessidade de intervenção governamental e ações de saúde voltadas ao cuidado da população de Barão de Cocais, principalmente relacionadas ao adoecimento mental. Além disso, subsidiam o estabelecimento de diretrizes na formulação de políticas públicas e tomadas de decisão dos órgãos governamentais e da mineradora, com o intuito de reforçar o apoio à população de Barão de Cocais em situação de vulnerabilidade social, além de mitigar os efeitos negativos decorrentes do risco de colapso da barragem.
